# Prognostic Value of Cancer Stem Cells Markers in Triple-Negative Breast Cancer

**DOI:** 10.1155/2015/158682

**Published:** 2015-10-04

**Authors:** Francesca Collina, Maurizio Di Bonito, Valeria Li Bergolis, Michelino De Laurentiis, Carlo Vitagliano, Margherita Cerrone, Francesco Nuzzo, Monica Cantile, Gerardo Botti

**Affiliations:** ^1^Pathology Unit, Istituto Nazionale Tumori Fondazione “G. Pascale”, IRCCS, 80131 Napoli, Italy; ^2^Department of Breast Surgery and Cancer Prevention, Istituto Nazionale Tumori Fondazione “G. Pascale”, IRCCS, 80131 Napoli, Italy

## Abstract

Triple-negative breast cancer (TNBC) has a significant clinical relevance of being associated with a shorter median time to relapse and death and does not respond to endocrine therapy or other available targeted agents. Increased aggressiveness of this tumor, as well as resistance to standard drug therapies, may be associated with the presence of stem cell populations within the tumor. Several stemness markers have been described for the various histological subtypes of breast cancer, such as CD44, CD24, CD133, ALDH1, and ABCG2. The role of these markers in breast cancer is not clear yet and above all there are conflicting opinions about their real prognostic value. To investigate the role of CSCs markers in TNBC cancerogenesis and tumor progression, we selected 160 TNBCs samples on which we detected protein expression of CD44, CD24, CD133, ALDH1, and ABCG2 by immunohistochemistry. Our results highlighted a real prognostic role only for CD44 in TNBCs. All other CSCs markers do not appear to be related to the survival of TNBC patients. In conclusion, despite the fact that the presence of the cancer stem cells in the tumor provides important information on its potential aggressiveness, today their detection by immunohistochemistry is not sufficient to confirm their role in carcinogenesis, because specific markers probably are not yet identified.

## 1. Introduction

Triple-negative breast cancer (TNBC) (tumors that do not express estrogen receptor (ER) and progesterone receptor (PR) genes and with nonoverexpressed/nonamplified HER-2 gene) accounts for 10%–24% of invasive breast cancers, and it is typically high-grade tumor with different histological types. The TNBC occurs predominantly in young African or African American women in premenopausal period and tends to display aggressive behavior demonstrating a great propensity to metastasize. The main metastatic locations are the bones and the central nervous system [1, 2]. Usually, patients with TNBC tend to have a higher recurrence rate after diagnosis, a short disease-free interval, and reduced overall survival, especially for the lack of targeted therapies [3]. Originally, several studies have shown that TNBC can be grouped into two main immunophenotypically and clinically distinct subgroups: (I) basal-like subtype that accounts for approximately 70% of TNBCs (expressing basal markers) and (II) the nonbasal subtype [4, 5].

Recently, Lehmann et al., by gene expression profiles studies, have further stratified the TNBCs into 6 subtypes, expressing many different molecular markers specific for the different groups [6]. However, more recently, another RNA and DNA genomic profiles study showed that TNBCs can be divided into four fundamental subtypes with molecular characteristics even more specific, often targets of biological therapies, with differential potentiality of aggressiveness [7]. In both studies, the molecular more aggressive subtypes were those associated with the expression of immunomodulatory and stem-like molecules.

Recent acquisitions on human carcinogenesis suggest that small populations of tumor stem cells can influence and modify neoplastic cells behavior and aggressiveness as well as therapeutic response. Many observations suggest that breast cancer ability to proliferate, progress, and spread is also based on a limited subpopulation of cells with properties similar to stem cells, defined as “breast cancer stem cells” (BCSCs) [8, 9].

Several stemness markers have been described for identification of BCSCs in cancer subtypes, such as CD44, CD24, CD133, EpCAM, CD166, Lgr5, CD47, ALDH1, and ABCG2 [9, 10].

CD44/CD24 expression profiles showed a large variability within breast cancer subtypes [11] especially for TNBCs. In fact, Idowu et al. [12] showed that CD44^+^CD24^−/low^ phenotype was associated with a worse prognosis in TNBCs patients, while Giatromanolaki et al. [13] described that CD44^−^CD24^−^ phenotype was associated with a worse prognosis also in TNBCs. Finally, Ahmed et al. observed that CD44^−^CD24^+^ phenotype was the only one associated with poor prognosis in breast cancer [14].

Other studies suggested that ABCG2 alone can be considered a suitable marker for breast cancer, in particular for TNBC phenotype, but this observation was limited to cellular models [15]. ALDH1 expression was described to be higher in TNBC than non-TNBC cell [16], and in a small case series of TNBC patients its expression was associated with poor clinical outcomes [17].

Recently, CD133 proved to be suitable also in the identification of CSCs in TNBC, as shown in several in vitro [18, 19] and in vivo studies [20]. In addition, the recent use of CD133 to detect circulating tumor cells in TNBC patients [21, 22] has increased attention to this marker highlighting its role in establishing prognostic and predictive value in TNBCs.

However, the role of these markers in breast cancer progression is not clearly defined and, above in TNBC phenotype, the most suitable for the characterization of the niches of tumor stem cells have not been determined. Most studies, in fact, were carried out on small series of TNBCs or on cellular models [15, 17, 20] and aimed at understanding the molecular mechanisms related to the single markers.

In this study we analyzed protein expression of CD133, CD24, CD44, ABCG2, and ALDH1 in a case series of TNBCs, included in a Tissue Microarray, to correlate their expression to clinic-pathological features and survival of TNBC patients and identify the CSCs marker with the best prognostic value.

## 2. Materials and Methods

### 2.1. Patients and Specimens

One hundred sixty patients who underwent mastectomy or quadrantectomy from 2003 to 2009 at the National Cancer Institute “Giovanni Pascale” of Naples were enrolled in this study. All cases were reviewed according to WHO classification criteria, using standard tissue sections and appropriate immunohistochemical slides.

Medical records were reviewed for clinical information; histologic parameters were determined from the H&E-stained slides. Clinicopathologic parameters evaluated for each tumor included patient age at initial diagnosis, tumor size, histologic subtype, nuclear grade, number of positive lymph nodes, tumor stage, tumor recurrence, distant metastasis, and type of surgery. Moreover, all specimens were characterized for all routine diagnostic immunophenotypic parameters.

### 2.2. TMA Building

Tissue Microarray (TMA) was built using the most representative areas from each single case with one replicate. All tumours and controls were reviewed by two experienced pathologists (MDB/GB). Discrepancies between two pathologists from the same case were resolved in a joint analysis of the cases. Tissue cylinders with a diameter of 1 mm were punched from morphologically representative tissue areas of each “donor” tissue block and brought into one recipient paraffin block (3 × 2.5 cm) using a semiautomated tissue arrayer (Galileo TMA).

### 2.3. Immunohistochemistry Analysis

Before the preparation of the TMA on whole sections breast tumor samples were characterized for routinely immunophenotypical parameters, including ER, PgR, HER2, and Ki67. All samples which were negative for ER, PgR, and ErbB2 (TNBCs subtype) were included in the study. To confirm the diagnosis, all three markers were again analyzed on TMA slides.

Immunohistochemical staining was done on 9 TMA slides from formalin-fixed, paraffin embedded tissues to evaluate the expression of CD133, ER, PgR, c-erbB2, Ki67, CD24, CD44, ALDH1A1, and ABCG2 markers. Paraffin slides were then deparaffinized in xylene and rehydrated through graded alcohols. Antigen retrieval was performed with slides heated in 0.01** **M citrate buffer (pH 6.0 for CD133, ABCG2, PgR, c-erbB2, Ki67, CD24, and CD44) or Tris-EDTA (pH 9 for ER and ALDH) in a bath for 20** **min at 97°C. After antigen retrieval, the slides were allowed to cool. The slides were rinsed with TBS and the endogenous peroxidase was inactivated with 3% hydrogen peroxide. After protein block (BSA 5% in PBS 1x), the slides were incubated with primary antibody to human CD133 (Miltenyi Biotec Monoclonal Mouse CD133/1 (AC 133) pure 1 : 150) and CD24 (Abcam Rabbit Polyclonal Anti-CD24 ab110448 1 : 100) for one hour, to human ER*α* (DAKO Monoclonal Mouse Anti-Human ER*α* Clone ID5 1 : 35), PR (DAKO Monoclonal Mouse Anti-Human PR Clone 636 1 : 50), c-erbB2 (DAKO Polyclonal Rabbit Anti-Human Oncoprotein 1 : 300), Ki67 (DAKO Monoclonal Mouse Anti-Human Ki67 Ab Clone MIB-1 1 : 75), CD44 (Novocastra Lyophilized Mouse Monoclonal Antibody CD44 Variant 3 1 : 35) for 30 minutes, and to ABCG2 (Abcam Mouse Monoclonal Anti-BCRP/ABCG2 antibody ab3380 1 : 30) and ALDH1A1 (Abcam Rabbit Monoclonal Anti-ALDH1A1 antibody (ab52492), 1 : 100) overnight. The sections were rinsed in TBS and incubated for 20** **min with Novocastra Biotinylated Secondary Antibody (RE7103), a biotin-conjugated secondary antibody formulation that recognized mouse and rabbit immunoglobulins. Then the sections were rinsed in TBS and incubated for 20** **min with Novocastra Streptavidin-HRP (RE7104) and then peroxidase reactivity was visualized using a 3,3′-diaminobenzidine (DAB). Finally, the sections were counterstained with hematoxylin and mounted. Results are interpreted using a light microscope.

### 2.4. Evaluation of Immunohistochemistry

Antigen expression was evaluated independently by two pathologists using light microscopy. Observer was unaware of the clinical outcome. For each sample, two cores (inside the tumor) were analyzed. Using a semiquantitative scoring system microscopically and referring to each antigen scoring method in other studies, an observer evaluated the intensity, extent, and subcellular distribution of CD133, ER, PR, c-erbB2, Ki67, ABCG2, ALDH1A1, CD24, and CD44. The cutoff used to distinguish “positive” from “negative” cases was ≥1% ER/PR positive tumor cells. Immunohistochemical analyses of c-erbB2 expression describe the intensity and staining pattern of tumor cells. Only membrane staining intensity and pattern were evaluated using the 0 to 3+ score as illustrated in the HercepTest kit scoring guidelines. The ASCO/CAP 2013 describes a new HER2 Testing Algorithms identifying 4 categories: no staining or incomplete and faint/barely perceptible membrane staining within ≤10% of tumor cells (0 negative); incomplete and faint/barely perceptible membrane staining within >10% of tumor (1+ negative); incomplete and circumferential weak/moderate membrane staining within >10% of tumor cells or complete and circumferential intense membrane staining within ≤10% of tumor cells (2+ equivocal); and complete and circumferential intense membrane staining within >10% of tumor cells (3+ positive). Cases with score 2+ underwent fluorescence in situ hybridization analysis. The proliferative index Ki67 was defined as the percentage of immunoreactive tumour cells out of the total number of cells (low = ≤20%; high = >20%). In scoring CD133, CD44, and ABCG2 proteins expression, both the extent and intensity of immunopositivity in the cell membrane and cytoplasm were considered, while, for CD24 and ALDHA1, we have considered only the cytoplasmic staining.

There are not standardized criteria for CD133, CD44, CD24, and ALDHA1 markers staining evaluation; thus we schematized our score evaluation as follows: for CD133 we considered the positivity or negativity of the staining; for CD24 staining we evaluated cell percentage positivity (low = <50%/high = ≥50%); for ALDHA1 staining we evaluated cell percentage positivity (low = <25%/high = ≥25%); for cytoplasmic CD44 we considered the expression as high when the cell positivity percentage was >50% with intermediate-high intensity and considered the expression as low when it was ≤50%; for membrane CD44 we considered the expression as high when the cell positivity percentage was ≥25% with intermediate-high intensity and considered the expression as low when it was <25%.

The ABCG2 score was determined by combining the proportion of positively stained tumor cells and the intensity of staining as previously described [23].

### 2.5. Statistical Analysis

The association between CD133, CD44, CD24, ALDH, and ABCG2 with each other and with the clinicopathological data was conducted using *χ* ^2^ or Spearman correlation test when appropriate. Pearson's *χ* ^2^ test was used to determine whether a relationship exists between the variables included in the study. The level of significance was defined as *p* < 0.05. Overall Survival (OS) and Disease-Free Survival (DFS) curves were calculated using Kaplan-Meier method.

OS was defined as the time from diagnosis (first biopsy) to death by any cause or until the most recent follow-up. DFS was measured as the time from diagnosis to the occurrence of progression, relapse after complete remission, or death from any cause.

All the statistical analyses were carried out using the Statistical Package for Social Science v. 20 software (SPSS Inc., Chicago, IL, USA).

## 3. Results

### 3.1. Clinicopathological Characteristics of TNBC Patients

In our cohort, we have included 160 TNBC samples of breast cancers 12 lobular, 4 mixed, 9 medullary, 1 mucinous, 6 metaplastic, and 128 invasive ductal breast carcinomas (including 5 TNBC metastases).

The age of patients ranged from 24 to 93 years, with an average age of 57 years. Tumor sizes were lower than 2 cm in 47.1% (73/155) of the samples, between 2 and 5 cm in 44.5% (69/155) of the samples, and larger than 5 cm in 8.4% (13/155) of the samples. These data were not available for the five cases of metastases that have been included in the study. Metastatic lymph nodes were found in 43.1% (66/153) of patients at surgery (this information for 7 patients was lost), while distant metastases were found in 24.4% (39/155). 5 cases were unable to recover this information. The percentages of tumor grading were 86.5% (134/155) grade 3, 12.2% (19/155) grade 2, and 1.3% (2/155) grade 1. The expression of proliferation factor Ki67 was high (>20%) in 121/153 cases (79.1%) and low (≤20%) in 32/153 cases (20.9%). This information for 7 patients was lost. All clinicopathological characteristics are shown in Tables 1 and 2.

### 3.2. CD44 Expression in TNBC Patients

**Figure 1:**
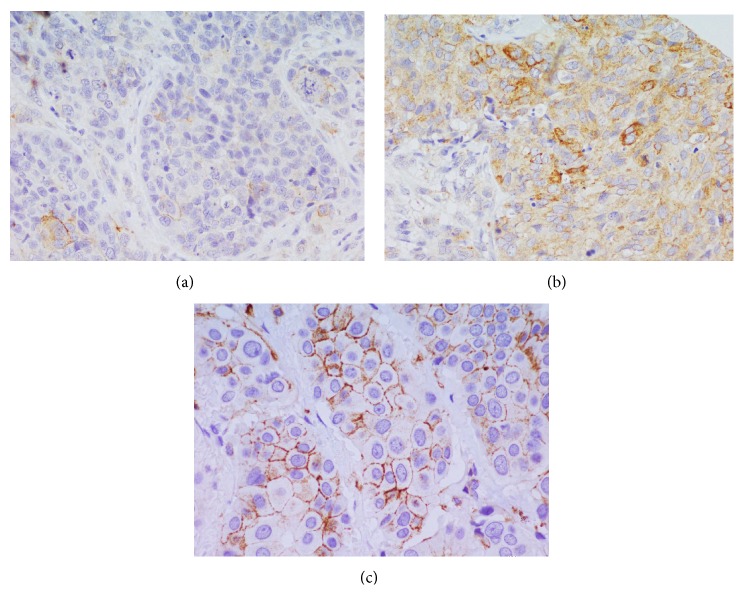
CD44 immunostaining in TNBC. (a) Low membrane and cytoplasmic expression (40x). (b) High membrane and cytoplasmic expression (40x). (c) High membrane expression (40x).

CD44 protein expression was detected, excluding the samples that could not be assessed, in 143/160 samples. In 108 samples, there was a low cytoplasmic CD44 expression, while, in 35 samples, there was a high expression (Figure 1). The membrane expression was low in 133 cases and high in 10 cases, while only 4/143 cases showed cytoplasmic and membrane expression.

**Figure 2:**
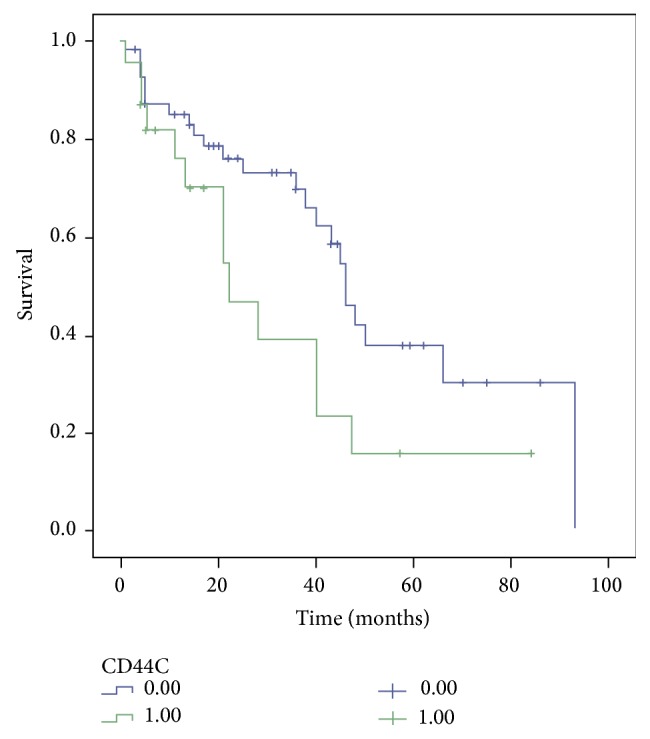
CD44 Disease-Free Survival Kaplan-Meier curve. The patients with high expression of cytoplasmic CD44 have worse prognosis than those with low expression of CD44.

Based on statistical elaboration of CD44 protein expression analysis with the other clinicopathological parameters in TNBC, considering only cytoplasmic expression, we showed that CD44 was significantly associated with metastases (*p* = 0.011) (Table 1) and with DFS (*p* = 0.051) (Figure 2). No statistical association with OS was present. Moreover, there was a trend of statistical association with proliferation index Ki67 (*p* = 0.078).

If we consider only membrane positivity, a trend of inverse association with distant metastases (*p* = 0.085) was present (Table 1). Considering membrane and cytoplasmic positive immunostaining, a direct association with age of patients (>40 ≤60) (*p* = 0.051) and a trend of inverse association with lymph node metastases were present (*p* = 0.087) (Table 1). In these cases, there were no statistical associations with DFS (*p* = 0.462 and *p* = 0.609, resp.) or OS.

### 3.3. CD133 Expression in TNBC Patients

**Figure 3:**
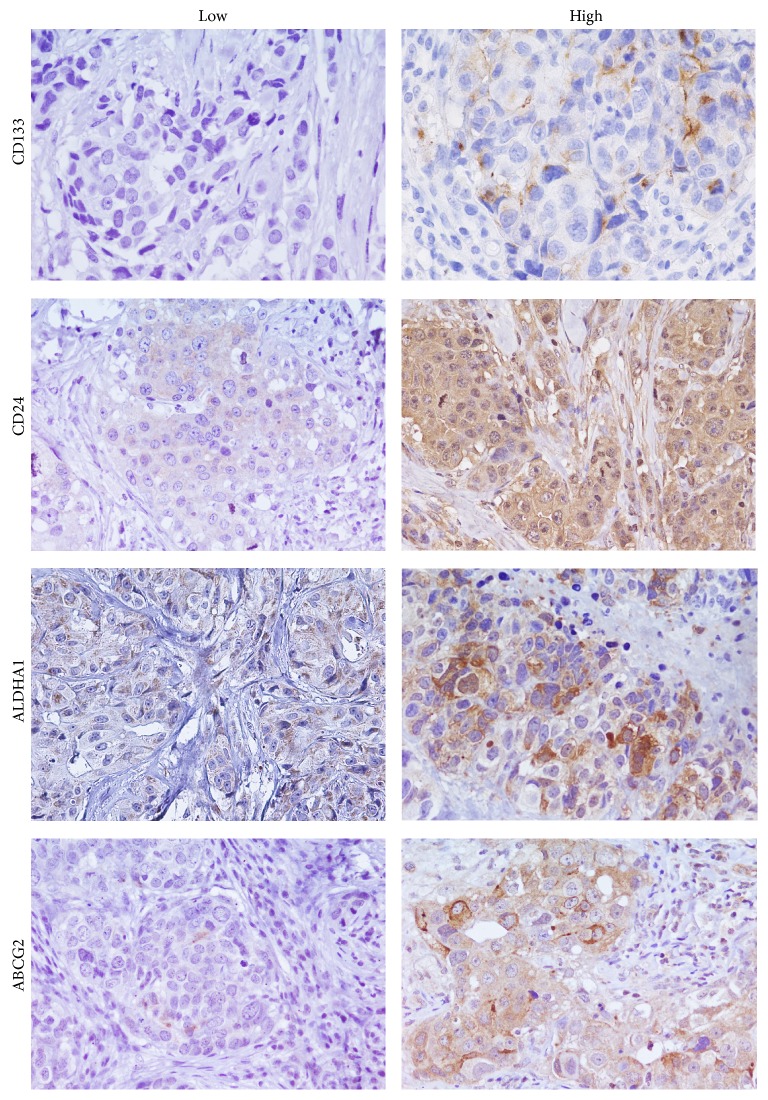
CD133, CD24, ALDH1A1, and ABCG2 immunostaining in TNBC (40x).

146/160 samples were stained for CD133. 116/146 samples were negative and 30/146 samples were positive (Figure 3). 14/160 samples are missing data.

Based on statistical elaboration of CD133 protein expression analysis with the clinicopathological parameters in TNBCs, we showed that only a trend of statistical association with the ductal histotype (*p* = 0.088) (Table 1) was present. No statistical association with OS and DFS was present.

### 3.4. CD24 Expression in TNBC Patients

CD24 protein expression was detected, excluding the samples that could not be assessed, in 137/160 samples. In 123 samples there was a high CD24 expression; in 14 samples there was a low expression (Figure 3).

Statistical analysis showed only a direct association with invasive ductal histotype (*p* = 0.059) (Table 2). No statistical association with DFS and OS was present.

### 3.5. ABCG2 Expression in TNBC Patients

ABCG2 protein expression was detected, excluding the samples that could not be assessed, in 141/160 samples. In 88 cases there was a high ABCG2 expression; in 27 cases there was a low expression and 26 cases were negative (Figure 3).

Statistical analysis showed a significant association only with proliferative index Ki67 (*p* = 0.024) (Table 2). No statistical association with DFS was present. An inverse trend of statistical association with OS was present (*p* = 0.081) (see Figure S1 in Supplementary Material available online at http://dx.doi.org/10.1155/2015/158682).

### 3.6. ALDHA1 Expression in TNBC Patients

ALDHA1 protein expression was detected, excluding the samples that could not be assessed, in 145/160 samples. In 45 cases there was a high ALDHA1 expression; in 100 cases there was a low expression (Figure 3).

Following the statistical analysis, a direct statistical association with Ki67 was found (*p* = 0.042) (Table 2). No statistical association with DFS and OS was present.

### 3.7. Relation between All CSCs Markers

Statistical analysis showed no significant associations between all cancer stem cell markers considered (data not shown).

## 4. Discussion

Breast tumors are a heterogeneous group of malignancies, which differ in morphology, gene profiling, prognosis, and therapeutic response [24].

Immunophenotypic analysis identify a particular breast tumor subtype, defined TNBC, because it do not express ER and PgR hormone receptors and shows non-overxepressed/amplified HER-2 oncogene. Its incidence is particularly high in younger women and the clinical course of the disease is often very aggressive [1, 2].

For this reason, the search for new molecular markers that can better explain the biology of this disease and especially its progression is becoming essential for the development of new and more appropriate therapeutic strategies.

Recently, the identification of cancer stem cell niches in tumor tissues is acquiring a great prognostic value, mainly because breast carcinogenesis may be the result of deregulation of molecular pathways controlling self-renewal of mammary epithelial cells [9].

Numerous cell surface markers have been used for the identification of stem cell clones in several tumors, but, for breast cancers, CSCs detection appears much more complex because of the extreme heterogeneity of histotypes/phenotypes that characterize these tumors [24].

The main objective of this study was the use of a panel of stem cell markers, selected on the basis of the recent experimental evidences found in literature, to evaluate their expression in TNBCs and verify their potential prognostic value.

Primarily, our data showed a very heterogeneous distribution of the selected markers expression. CD24 showed an association with IDC histotype and a strong statistical association with proliferation index ki67 but no association with patients' survival. Review of the literature shows that the role of CD24 in breast cancer and specifically in TNBC has been extensively investigated.

However, the evaluation of this marker has always been associated with the prognosis combined to the CD44 expression [11–13]. In our study, the combination of the two markers showed no association with the survival of TNBCs patients.

Regarding ABCG2, its expression appeared very high in most of TNBCs and also was the only marker which showed a strong association with Ki67.

In literature, there are no data on the expression of ABCG2 in TNBCs samples as they exist only on cellular models as MDA-MB-231 cell lines [15]. Thus, our data showed for the first time the expression of ABCG2 on a cohort of TNBCs patients.

ALDHA1 appeared also expressed in the majority of TNBCs but its overexpression was detected only in 28% of cases. This marker showed only an association with Ki67, while no association with other clinicopathological features and survival of TNBCs patients were highlighted.

These observations appeared to contrast with those reported in the literature, where ALDHA1 is described as an independent prognostic factor in TNBC patients [25]. Ohi et al. showed ALDH1 expression in 51% of TNBC cases with a heterogeneous immunoreactivity in the cytoplasm of carcinoma cells as well as in macrophages, stromal fibroblasts, peripheral nerves, and vascular smooth muscle cells. We detected ALDH1 only in tumor cells and in some TNBC samples. We suppose that discordance in the ALDH1 staining can be associated with the different clones of antibodies used for staining and the subjective definition of the IHC score for evaluation.

CD133 expression in TNBC patients was detected only in 20% of cases. Its expression appeared to be associated with ductal histotype but it showed no statistical association with other clinicopathological parameters and survival.

Recent and numerous studies showed that positivity for CD133 allows identifying CSCs in breast cancer [26]. CD133 is expressed by several solid tumors, including invasive TNBC, with very low levels of expression compared to other CSCs markers previously reported, like CD44 and ALDH1 [27]. In early-onset breast cancers, associated with mutations on BRCA1, CD133+ cells show CSCs properties [26]. The employment of this tumour stemness marker in breast cancers has become popular more recently and its expression is often described as being associated with a worse prognosis [20, 28]. In TNBCs patients the role of CD133 was previously documented, showing that this marker expression was correlated with prognosis [28].

However, our data showed no strong statistical association with TNBCs patients' survival in contrast to all the experimental evidences in the literature. Zhao et al. investigated CD133 expression in 67 TNBCs patients showing its expression in 43.3% of cases with a predominant expression in the membrane and minimally in the cytoplasm of the tumor cell. We described CD133 positivity in 20% of samples and with a prevalent cytoplasmic expression. Even in this case the discordant date may be associated with the different clones of antibodies used for staining and the subjective definition of the IHC score for evaluation. In fact, for all of these markers, there are not standardized criteria of evaluation that make doubt their real prognostic value.

Finally, regarding CD44 protein expression, detection revealed a heterogeneous distribution of membranous end cytoplasmic positivity, which we have separately correlated with clinicopathological parameters and survival of patients. The results seem to be opposite to cytoplasmic staining strongly correlated with metastasis and disease-free survival, while the membrane staining showed a trend of inverse association with metastases. Cytoplasmic positivity for CD44 may be associated with its cytoplasmic domain [29] and it can be considered as an independent parameter during cancer progression [30, 31], although in this case the association with Ki67 appears to be evident.

Moreover, during normal cell physiology, several isoforms of CD44, by alternatively splicing, can be generated. The mechanisms by which CD44 controls signaling events are not clear. Many evidences showed that CD44 is assembled in a regulated manner into membrane-cytoskeletal junctional complexes and, through both direct and indirect interactions, serves to focus on downstream signal transduction events [32]. The role of CD44 in breast cancer was abundantly described in literature, in particular its duality in cancer progression [29]. Some studies showed a protumorigenic role for CD44 [33], while others showed a protective role for CD44 in breast cancer [34], suggesting that CD44 may influence tumor growth or metastasis differently at different phases of tumor progression. Variability in CD44-mediated biology could be due to the expression of alternatively spliced isoforms. In fact, the expression of CD44 variants has produced conflicting results with no definitive association between expression and clinical outcomes [29].

For identification of stem cell phenotype, many studies showed that high levels of CD44 associated to low levels of CD24 (CD44(+)/CD24(−/low)) would characterize stem populations in breast cancer [12]. However, this acquisition is not sufficiently clarified at least in breast cancer disease, where the combination of expression between the two markers can be very variable [11–14]. Our data on TNBCs suggest that their expressions would not be sufficiently selective for the identification of CSCs and their prognostic value contrasts with that reported in the literature [11–14]. CD44 alone seems to be a potential prognostic marker being statistically associated with the DFS of patients when its expression was cytoplasmic, while CD24low/CD44high not highligthed a prognostic role for TNBCs.

In conclusion, our data showed that all CSC markers selected seem to be associated only with the proliferative index in TNBCs, while the only marker significantly associated with the prognosis of TNBCs was CD44.

Despite the fact that the prognostic value of these markers has been thoroughly described in breast cancer, probably the use of these markers by immunohistochemistry not only fails to identify niches of stem cells, showing an abundant and heterogeneous expression in tumor samples, but also does not seem to have a real prognostic value in TNBC.

This could be due to the limit of the technique and the extreme heterogeneity and specificity of commercial antibodies and could also be due to TNBCs being no longer a homogeneous tumor class. Lehmann et al., by gene expression profiles studies, have further stratified the TNBCs into 6 subtypes, expressing many different molecular markers specific for the different groups [6]. However, more recently, another genomic profiles study showed that TNBCs can be divided into four fundamental subtypes with molecular characteristics even more specific, often targets of biological therapies, with differential potentiality of aggressiveness [7].

In both studies, Basal-Like Immune Activated (BLIA) (with upregulation of genes controlling B cell, T cell, natural killer cell functions and inflammatory cytokines) and mesenchymal stem like (MSL) subtypes represent the more aggressive molecular subtypes.

Perhaps these markers may manifest a stronger prognostic value if we were capable of subtyping TNBCs evaluating their expression in MSL subtype.

In conclusion, our data supported the idea that it is necessary to identify more specific CSCs markers for prognostic stratification of TNBCs by immunohistochemistry.

## Supplementary Material

Overall Survival Kaplan-Meier curve. The patients with ABCG2 high expression have better prognosis than those with ABCG2 low expression.

## Figures and Tables

**Table 1:** Relation between CD133 and CD44 (cytoplasmic, membranous, and cytoplasmic/membranous positivity) markers with clinical pathological features in TNBC patients.

	CD133		*p* value	CD44C		*p* value	CD44M		*p* value	CD44CM		*p* value
	Negative	Positive		Low	High		Low	High		Low	High	
Age												
<40	11 (63,1%)	4 (36,9%)	0,806	15 (78,9%)	4 (21,1%)	0,783	18 (94,7%)	1 (5,3%)	0,440	19 (100%)	0 (0%)	0,051
>40 ≤ 60	49 (84,6%)	13 (15,4%)		42 (72,4%)	16 (27,6%)		52 (89,7%)	6 (10,3%)		54 (93,1%)	4 (6,9%)	
>60	55 (75,7%)	13 (24,3%)		50 (80,6%)	15 (19,4%)		62 (95,4%)	3 (4,6%)		65 (100%)	0 (0%)	
Missed data	1	0		1	0		1	0		1	0	
Histotype												
IDC	93 (76,9%)	28 (23,1%)	0,088	88 (75,2%)	29 (24,8%)	0,855	107 (91,5%)	10 (8,5%)		114 (97,4%)	3 (2,6%)	0,720
NIDC	23 (92%)	2 (8%)		20 (76,9%)	6 (23,1%)		26 (100%)	0 (0%)	0,122	25 (96,2%)	1 (3,8%)	
Missed data	0	0		0	0		0	0		0	0	
Size												
≤2 cm	55 (79,7%)	14 (20,3%)	0,934	48 (75%)	16 (25%)	0,768	60 (93,8%)	4 (6,2%)	0,408	63 (98,4%)	1 (1,6%)	0,470
>2 ≤ 5	49 (79%)	13 (21%)		47 (73,4%)	17 (26,6%)		60 (93,8%)	4 (6,2%)		61 (95,3%)	3 (4,7%)	
>5	9 (75%)	3 (25%)		10 (83,3%)	2 (16,7%)		10 (83,3%)	2 (16,7%)		12 (100%)	0 (0%)	
Missed data	3	0		3	0		3	0		3	0	
LNM												
Negative	63 (80,5%)	19 (19,5%)	0,517	62 (77,5%)	18 (22,5%)	0,333	73 (91,3%)	7 (8,7%)	0,439	76 (95%)	4 (5%)	0,087
Positive	48 (90,6%)	11 (9,4%)		40 (70,2%)	17 (29,8%)		54 (94,7%)	3 (5,3%)		57 (100%)	0 (0%)	
Missed data	5	0		6	0		6	0		6	0	
Metastasis												
Negative	89 (77,4%)	26 (25,6%)	0,235	90 (80,4%)	22 (19,8%)	0,011	102 (91%)	10 (9%)	0,085	108 (96,4%)	4 (3,6%)	0,286
Positive	27 (87%)	4 (13%)		18 (58,1%)	13 (41,9%)		31 (100%)	0 (0%)		31 (100%)	0 (42%)	
Missed data	0	0		0	0		0	0		0	0	
Grading												
G1	2 (100%)	0 (0%)	0,223	1 (100%)	0 (0%)	0,844	1 (100%)	0 (0%)	0,463	1 (100%)	0 (0%)	0,751
G2	15 (93,7%)	1 (6,3%)		12 (75%)	4 (25%)		16 (100%)	0 (0%)		16 (100%)	0 (0%)	
G3	96 (76,8%)	29 (23,2%)		91 (74,6%)	31 (25,4%)		112 (91,8%)	10 (8,2%)		118 (96,7%)	4 (3,3%)	
Missed data	3	0		4	0		4	0		4	0	
Ki67												
≤20%	24 (85,7%)	4 (14,3%)	0,358	23 (92%)	3 (8%)	0,078	24 (92,3%)	2 (7,7%)	0,941	26 (100%)	0 (0%)	0,324
>20%	88 (77,9%)	25 (22,1%)		79 (71,8%)	31 (28,2%)		102 (92,7%)	8 (7,3%)		106 (96,4%)	4 (3,6%)	
Missed data	4	1		6	1		6	0		7	0	

IDC = infiltrant ductal carcinoma; NIDC = noninfiltrant ductal carcinoma; LNM = lymph node metastasis.

**Table 2:** Relation between CD24, ALD1A1, and ABCG2 with clinical pathological features in TNBC patients.

	CD24		*P* value	ALDH		*P* value	ABCG2			*P* value
	Low	High		Low	High		Negative	Low	High	
Age										
<40	1 (5,6%)	17 (94,4%)		13 (68,4%)	6 (28,6%)		2 (11,1%)	7 (38,9%)	9 (50%)	
>40 ≤ 60	7 (12,3%)	50 (87,7%)	0,706	39 (65%)	21 (35%)	0,559	9 (15,8%)	8 (14%)	40 (70,2%)	0,132
>60	6 (9,8%)	55 (90,2%)		48 (73,8%)	17 (26,2%)		15 (23%)	12 (18,5%)	38 (58,5%)	
Missed data	0	1		0	1		0	0	1	
Histotype										
IDC	9 (8%)	104 (92%)		85 (70,8%)	35 (29,2%)		23 (19,5%)	21 (17,8%)	74 (62,7%)	
NIDC	5 (20,8%)	19 (79,2%)	0,059	15 (60%)	10 (40%)	0,427	3 (13%)	6 (26%)	14 (61%)	0,567
Missed data	0	0		0	0		0	0	0	
Size										
≤2 cm	4 (6,6%)	57 (93,4%)		43 (67,2%)	21 (32,8%)		14 (21,5%)	12 (18,5%)	39 (60%)	
>2 ≤ 5	9 (14,8%)	52 (85,2%)	0,314	46 (69,7%)	20 (30,3%)	0,946	10 (16,1%)	10 (16,1%)	42 (67,8%)	0,599
>5	1 (7,7%)	12 (92,3%)		8 (66,6%)	4 (33,4%)		2 (16,6%)	4 (33,4%)	6 (50%)	
Missed data	0	2		3	0		0	1	1	
LNM										
Negative	8 (10,4%)	69 (89,6%)		58 (74,4%)	20 (25,6%)		13 (17,3%)	15 (20%)	47 (62,7%)	
Positive	6 (11,1%)	48 (88,9%)	0,895	38 (62,3%)	23 (37,7%)	0,127	13 (21,6%)	10 (16,7%)	37 (61,7%)	0,767
Missed data	0	6		4	2		0	2	4	
Metastasis										
Negative	11 (10,4%)	95 (89,6%)		80 (70,2%)	34 (29,8%)		20 (18,5%)	21 (19,4%)	67 (62%)	
Positive	3 (9,7%)	28 (90,3%)	0,910	20 (64,5%)	11 (35,5%)	0,546	6 (18,2%)	6 (18,2%)	21 (63,6%)	0,982
Missed data	0	0		0	0		0	0	0	
Grading										
G1	0 (0%)	2 (100%)		1 (100%)	0 (0%)		0 (0%)	1 (100%)	0 (0%)	
G2	1 (9%)	10 (91%)	0,875	7 (53,8%)	6 (46,2%)	0,386	4 (36,4%)	2 (18,2%)	5 (45,4%)	0,145
G3	13 (10,7%)	108 (89,3%)		89 (70,1%)	38 (29,9%)		22 (17,5%)	23 (18,2%)	81 (64,3%)	
Missed data	0	3		3	1		0	1	2	
Ki67										
≤20%	2 (8,3%)	22 (91,7%)		21 (91,3%)	3 (8,7%)		8 (32%)	7 (28%)	10 (40%)	
>20%	9 (8,5%)	97 (91,5%)	0,980	76 (66,7%)	38 (33,3%)	0,042	16 (14,4%)	19 (17,1%)	76 (68,5%)	0,024
Missed data	3	4		3	4		2	1	2	

IDC = infiltrant ductal carcinoma; NIDC = noninfiltrant ductal carcinoma; LNM = lymph node metastasis.
