# Supplementary figures and images for: Prognostic Value of Cancer Stem Cells Markers in Triple-Negative Breast Cancer
Source: Biomed Res Int. 2015 Oct 4;2015:158682. doi: 10.1155/2015/158682 (PMC4609334; doi:10.1155/2015/158682)

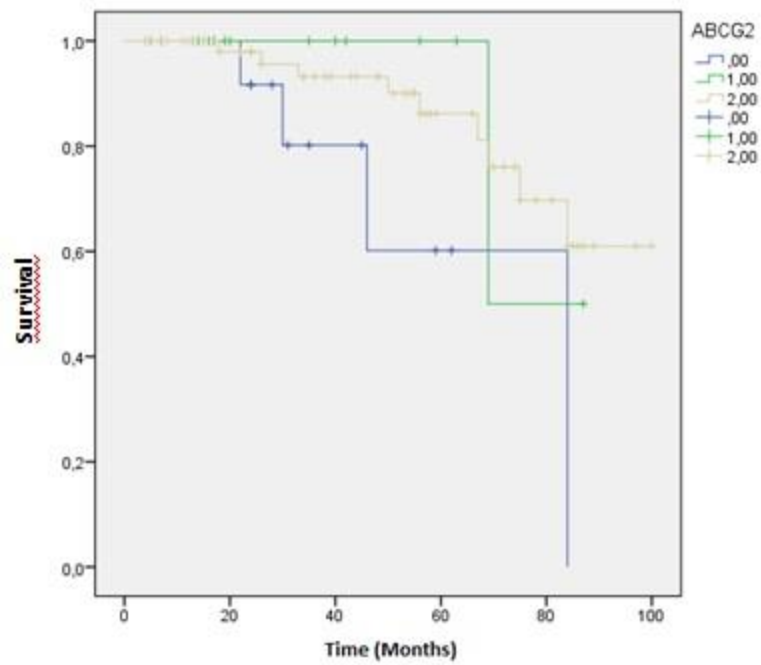

Supplementary Figure 1

Supplement: Supplementary file 1 — Overall Survival Kaplan-Meier curve. The patients with ABCG2 high expression have better prognosis than those with ABCG2 low expression. [file 158682.f1.pdf]
